# Electroconvulsive Therapy Added to Non-Clozapine Antipsychotic Medication for Treatment Resistant Schizophrenia: Meta-Analysis of Randomized Controlled Trials

**DOI:** 10.1371/journal.pone.0156510

**Published:** 2016-06-10

**Authors:** Wei Zheng, Xiao-Lan Cao, Gabor S. Ungvari, Ying-Qiang Xiang, Tong Guo, Zheng-Rong Liu, Yuan-Yuan Wang, Brent P. Forester, Stephen J. Seiner, Yu-Tao Xiang

**Affiliations:** 1 The Affiliated Brain Hospital of Guangzhou Medical University (Guangzhou Huiai Hospital), Guangzhou, China; 2 Department of Psychiatry, Chinese University of Hong Kong, Hong Kong SAR, China; 3 Shenzhen Key Laboratory for Psychological Healthcare & Shenzhen Institute of Mental Health, Shenzhen Kangning Hospital & Shenzhen Mental Health Center, Shenzhen, China; 4 School of Psychiatry & Clinical Neurosciences, University of Western Australia, Perth, Australia; 5 The University of Notre Dame Australia / Marian Centre, Perth, Australia; 6 The National Clinical Research Center for Mental Disorders & Beijing Anding Hospital, Capital Medical University, Beijing, China; 7 Mental Hospital of Guangzhou Civil Administration, Guangzhou, 510430, China; 8 Department of Epidemiology and Biostatistics, Faculty of Public Health, Wuhan University, Wuhan, Hubei province, China; 9 McLean Hospital Belmont, MA & Harvard Medical School, Department of Psychiatry, Boston, MA, United States of America; 10 Unit of Psychiatry, Faculty of Health Sciences, University of Macau, Macao SAR, China; Benito Menni Complejo Asistencial en Salud Mental, SPAIN

## Abstract

This meta-analysis of randomized controlled trials (RCTs) examined the efficacy and safety of the combination of electroconvulsive therapy (ECT) and antipsychotic medication (except for clozapine) versus the same antipsychotic monotherapy for treatment-resistant schizophrenia (TRS). Two independent investigators extracted data for a random effects meta-analysis and pre-specified subgroup and meta-regression analyses. Weighted and standard mean difference (WMD/SMD), risk ratio (RR) ±95% confidence intervals (CIs), number needed to treat (NNT), and number needed to harm (NNH) were calculated. Eleven studies (n = 818, duration = 10.2±5.5 weeks) were identified for meta-analysis. Adjunctive ECT was superior to antipsychotic monotherapy regarding (1) symptomatic improvement at last-observation endpoint with an SMD of -0.67 (p<0.00001; I^2^ = 62%), separating the two groups as early as weeks 1–2 with an SMD of -0.58 (p<0.00001; I^2^ = 0%); (2) study-defined response (RR = 1.48, p<0.0001) with an NNT of 6 (CI = 4–9) and remission rate (RR = 2.18, p = 0.0002) with an NNT of 8 (CI = 6–16); (3) PANSS positive and general symptom sub-scores at endpoint with a WMD between -3.48 to -1.32 (P = 0.01 to 0.009). Subgroup analyses were conducted comparing double blind/rater-masked vs. open RCTs, those with and without randomization details, and high quality (Jadad≥adadup analyses were Jadad<3) studies. The ECT-antipsychotic combination caused more headache (p = 0.02) with an NNH of 6 (CI = 4–11) and memory impairment (p = 0.001) with an NNH of 3 (CI = 2–5). The use of ECT to augment antipsychotic treatment (clozapine excepted) can be an effective treatment option for TRS, with increased frequency of self-reported memory impairment and headache.

**Trial registration:**

CRD42014006689 (PROSPERO).

## Introduction

Treatment-resistant schizophrenia (TRS) remains a great clinical challenge [1, 2]. Clozapine is the most efficacious antipsychotic drug for this population [2, 3], but many patients cannot tolerate clozapine due to its adverse drug reactions (ADRs) [3, 4]. Apart from agranulocytosis, clozapine may cause sedation, constipation, and/or an increased the risk of metabolic syndrome, thereby lowering treatment adherence [5–7]. Augmenting strategies, such as antipsychotic polypharmacy, adjunctive use of antidepressants, mood stabilizers, fatty acid supplements, glutamatergic agents and electroconvulsive therapy (ECT) [8–10], have been considered for TRS.

ECT has shown to be an effective and safe augmentation in TRS including clozapine-resistant schizophrenia [11, 12]. It has been recommended for TRS by the Task Force Report of the American Psychiatric Association on ECT [13]. A number of randomized controlled trials (RCTs) [14–24] compared the efficacy and safety of ECT combined with antipsychotic medication other than clozapine with the same antipsychotic monotherapy. So far, the results have been conflicting.

A systematic review of 22 RCTs concluded that adjunctive ECT added to any type of antipsychotics could produce symptomatic improvement in TRS [25]. In another review of 12 RCTs Tharyan et al. [26] suggested that ECT combined with all types of antipsychotics may be an effective treatment for schizophrenia.

To the best of our knowledge no meta-analyses on ECT added to non-clozapine antipsychotics for TRS have been published. The present meta-analysis of RCTs evaluates the efficacy and safety of ECT added to non-clozapine antipsychotic medications for TRS, comparing this combination to antipsychotic monotherapy. In addition to international literature sources, Chinese databases that are not usually reviewed in the international literature were also searched.

## Methods

This meta-analysis is based on the methodology recommended by the Cochrane Collaboration [27] and prepared according to the Preferred Reporting Items for Systematic Reviews and Meta-Analyses (PRISMA) statement [28] (S1 Fig).

### Selection criteria

According to the *PICOS* acronym, the following selection criteria were used: Participants (*P*): patients with schizophrenia according to any diagnostic criteria. Intervention (*I*): ECT added to non-clozapine antipsychotic medications. Comparison (*C*): the same non-clozapine antipsychotic monotherapy or combined with sham ECT. Outcomes (*O*): efficacy and safety. Study design (*S*): RCTs reporting the efficacy and safety of adjunctive ECT for schizophrenia. Case series, observational trials, non-randomized trials, and non-original research (reviews and meta-analyses) were excluded.

### Outcome parameters

Clinical outcomes were based on intent-to-treat (ITT) analysis, if provided. The primary outcome measure was endpoint symptomatic improvement measured by the change in total psychopathology of the Positive and Negative Syndrome Scale (PANSS) [29], or the Brief Psychiatric Rating Scale (BPRS) [30]. Key secondary outcomes included early (at 1 to 2 weeks) symptomatic improvement, PANSS positive, negative and general psychopathology sub-scores, response and remission defined by individual studies, patient-reported ADRs during the study period, and all-cause discontinuation.

### Search methods

Major English (PubMed, PsycINFO, Embase, Cochrane Library databases, the Cochrane Controlled Trials Register) and Chinese databases (WanFang Database, Chinese Biomedical Database and China Journal Net) were searched from their inception until November 10, 2015 for RCTs using the following search terms: Electroconvulsive/Electroconvulsive therapy, schizophrenia, randomized controlled trial, placebo, and trial. The keywords were used in combination with the Boolean operators AND, OR, and NOT. The search was supplemented by using the “related article” function. Reference lists of eligible studies and relevant review articles were hand-searched. Authors were contacted for unpublished data if necessary.

### Data extraction

Selection of studies, data extraction and synthesis and assessment of bias were conducted independently by two authors (WZ and XLC). All information was checked by another author (YQX). Inconsistencies were resolved by discussion.

### Assessment of risk of bias

The methodological quality of RCTs was assessed by risk of bias [31] rating the method of random sequence generation (selection bias), allocation concealment (selection bias), blinding of participants and personnel (performance bias), blinding of outcome assessment (detection bias), incomplete outcome data (attrition bias), selective reporting (reporting bias) and other biases. Each domain was rated as “high risk”, “unclear risk”, or “low risk” [31]. In addition, the Jadad scale (range: 0–5) was used to assess study quality in five domains: “randomization,” “double blinding,” “description withdrawals and dropouts,” “generation of random numbers,” and “allocation concealment” [32]. RCTs were classified as high-quality when their Jadad total score was ≥3 and low quality when their Jadad score was <3 [33]. The GRADE system (S1 Table) was used to rate the quality of primary and secondary outcomes of the meta-analysis [34, 35].

### Data synthesis and statistical analyses

In order to combine studies, the random effects model [36] was used in all cases with the aid of the Review Manager Version 5.3 software (http://www.cochrane.org). Weighted or standard mean difference (WMD/SMD) and risk ratio (RR) with 95% confidence intervals (CIs) were calculated for continuous and dichotomous data, respectively. When RR was significant (p<0.05), the number needed to treat (NNT) or number needed to harm (NNH) were calculated. All statistical differences were considered significant when p<0.05.

In case of I^2^≥50% for the effect of primary outcomes, a sensitivity analyses was conducted after removing the three studies [14, 19, 23] that had an outlying effect size of SMD>-1.0. Furthermore, 5 comparative subgroup analyses were also conducted to identify potential moderators or mediators. These subgroup analyses were (1) Chinese vs. non-Chinese studies; (2) double blind/rater-masked vs. non-blinded studies; (3) duration of treatment <12 vs. ≥12 weeks; (4) mean number of ECT sessions <9 ECTs vs. ≥9 (since the number of ECT sessions was reported inconsistently, the median split of the number of recommended ECT sessions according to the ECT guidelines for schizophrenia in China were used, i.e., 6 to 12 sessions [37]; (5) high vs. low quality studies (≥3 vs. <3); and (6) co-starting vs. augmenting with antipsychotic. Since 2 RCTs [21, 24] provided data only for key secondary outcomes, subgroup analyses of endpoint symptomatic improvement were conducted with the remaining 9 RCTs.

Additionally, a meta-regression analysis was performed involving sample size, trial duration, Jadad score, mean age, percentage of male patients and illness duration to identify potential moderators or mediators of the effect on endpoint symptomatic improvement using the STATA Version 12.0 software (http://www.stata.com).

Finally, funnel plots, Egger’s intercept [38], Duval and Tweedie's Trim-and-fill procedure [39], and Rosenthal's fail-safe method [40] were used with the Comprehensive Meta-Analysis Version 2 software (http://www.meta-analysis.com) to assess publication bias of the primary outcomes.

## Results

### Literature search

**Fig 1:**
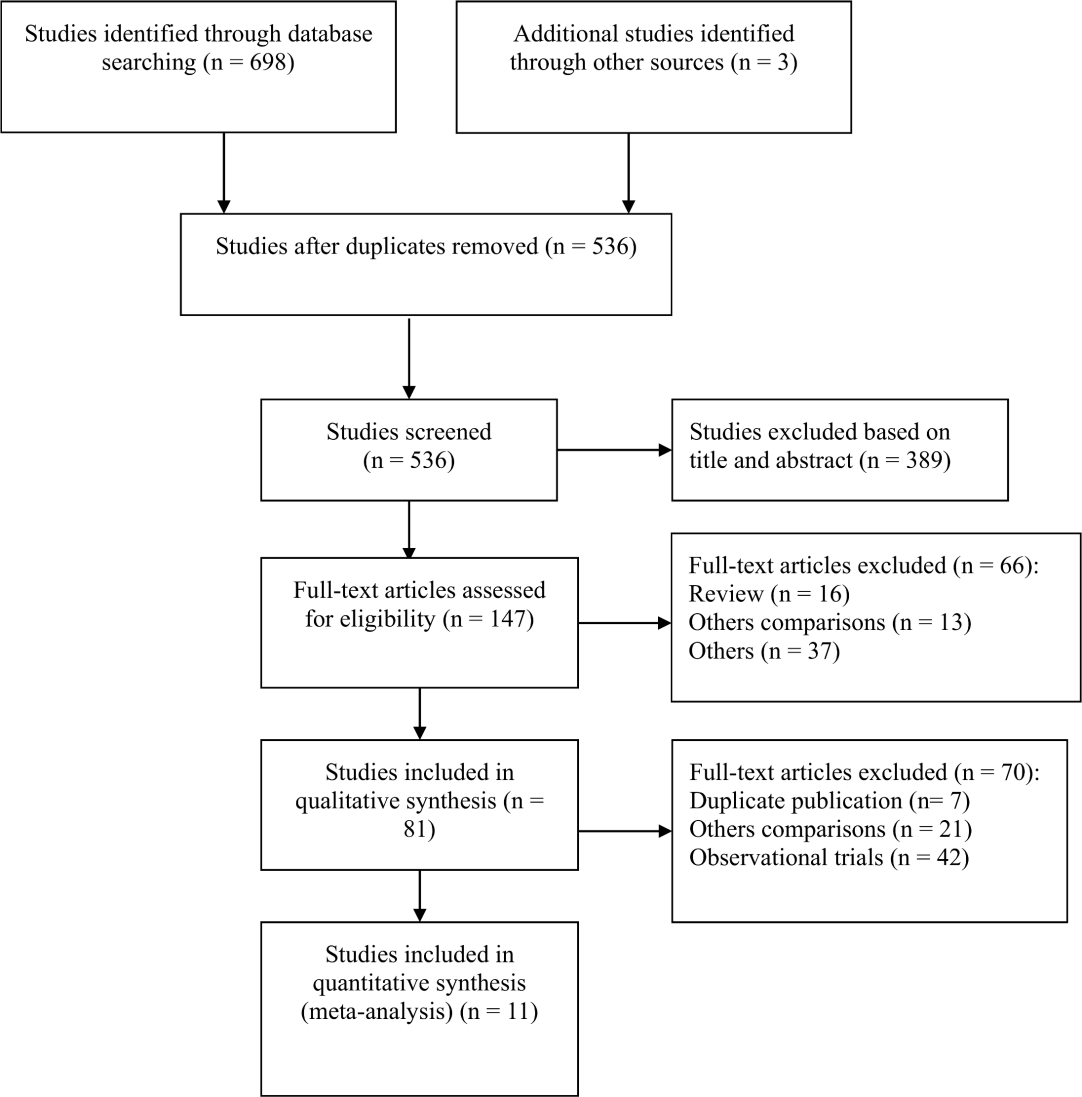
PRISMA flow diagram.

A total of 701 potentially relevant articles in the initial database search (698 trials) and other sources (3 trials) were ascertained. Eventually 11 RCTs met the selection criteria for meta-analysis (Table 1, Fig 1 and S2 Fig).

**Table 1:** ECT RCTs for schizophrenia and sample characteristics.

Author	Country	N^a^	Design				schizophrenia patients				ECT			Outcomes		Jadad score
			Duration (weeks)	Blinding	APs	Co-treatment^b^	Age (years)	Male (%)	Criteria	Refractory criteria	Bilateral/unilateral	Electricity dose	Section (times)	Psychotic scale	Cognition	
Chanpattana, 1999	Thailand	30	24	Open-label	Flu	Augmentation	34.9	43.3	NR	≥2 APs	Bilatera	346mC	14	BPRS	MMSE	3
Goswami, 2003	India	25	4	Double-blind	CPZ	Costart	29.5	64.0	DSM-IV	≥3 APs	Bilateral	NR	NR	BPRS	N/A	4
Jiang, 2009	China	67	12	Rater-masked	RIS	Augmentation	39.0	55.2	CCMD-3	≥3 APs	Bilateral	NR	12	PANSS	WCST	3
Li, 2015	China	160	8	Open-label	ZIP	Augmentation	34.5	62.5	DSM-IV	≥3 APs	NR	NR	12	BPRS	NR	2
Liu, 2012	China	65	12	Rater-masked	OLA	Costart	39.0	35.9	ICD-10	≥3 APs	Bilateral	188C	36	PANSS	NR	3
Wang, 2015	China	126	8	Open-label	ZIP	Augmentation	34.1	58.7	CCMD-3	≥3 APs	NR	NR	12	BPRS	WMS^C^	3
Wang, 2013	China	72	4	Open-label	OLA	Augmentation	45.5	59.7	CCMD-3	≥3 APs	Bilateral	20 Hz	10–12	PANSS	NR	2
Zhang, 2014	China	42	12	Open-label	OLA	Augmentation	35.5	69.1	CCMD-3	≥3 APs	NR	NR	8–12	PANSS	NR	3
Zhou, 2009	China	63	12	Open-label	OLA	Costart	42.6	36.5	CCMD-3	≥3 APs	Bilateral	NR	9.3	PANSS	WMS	2
Zhang, 2012	China	84	8	Rater-masked	OLA	Costart	38.4	44.0	CCMD-3	≥3 APs	NR	NR	16	PANSS	NR	3
Zhang, 2012	China	84	8	Open-label	QUE	Augmentation	34.1	59.5	CCMD-3	≥4 APs	Bilateral	NR	7.6	PANSS	NR	2

^a^This number reflects the total sample size recruited, including patients on RCTs.

^b^Co-treatment with ECT was started at the same time that other antipsychotic or added as an augmentation strategy.

^C^Data not provide for the control group

APs = antipsychotics; BPRS = Brief Psychiatric Rating Scale; CCMD-3 = China's mental disorder classification and diagnosis standard 3^th^ edition; CPZ = chlorpromazine; DSM-IV = Diagnostic and Statistical; ECT = electroconvulsive therapy; Flu = flupenthixol; ICD-10 = International Classification of Diseases, 10^th^ edition; MMSE = Mini-Mental Status Exam; NR = not report; OLA = olanzapine; PANSS = Positive and Negative Syndrome Scale; QUE = quetiapine; RIS = risperidone; ZIP = ziprasidone; WCST = Wisconsin Card Sorting Test; WMS = Wechsler Memory Scale

### RCTs and patient characteristics

Eleven RCTs with 818 patients (sample size range = 25–160, median = 67.0) compared add-on ECT (n = 414) to an antipsychotic medication, including chlorpromazine, flupenthixol, olanzapine, quetiapine, risperidone, and ziprasidone with the same antipsychotic monotherapy (n = 404). TRS was defined as failure to respond to ≥2 antipsychotics (1 trial), ≥3 antipsychotics (9 trials), and ≥4 antipsychotics (1 trial). Nine RCTs were conducted in China (n = 763), and 1 each in Thailand (n = 30) and India (n = 25). Only one RCT [15] used sham ECT in the control group.

Patients were 37.0±4.5 years (range = 29.5–45.5 years, median = 35.5 years), 55.4±10.0% were males (range = 36.5%-69.1%, median = 58.7%) and the mean illness duration was 13.0±4.3 years (range = 7.3–20.0 years, median = 12.5 years). The RCTs lasted 10.2±5.5 weeks (range = 4–24 weeks, median = 8.0 weeks). ECT courses comprised 14.2±8.0 sessions (range = 7.6–36.0 sessions, median = 8.0 sessions.

### Assessment of risk of bias

While 6 RCTs with a specific description regarding random sequence generation were rated as low risk, 6 RCTs were rated as high risk for allocation concealment. Masked assessors and double blindness were administered in 4 and 1 RCT, respectively. Regarding outcome data, 1 RCT reported loss to follow-up, but failed to use ITT analysis for incomplete outcome data. In addition, none of the studies registered their protocol, preventing formal assessment of reporting bias. Other biases were rated as low risk in all RCTs (S3 Fig).

### Quality assessment

The Jadad score was 2.6±0.7 (range = 2–4, median = 3) (Table 1); 7 RCTs were classified as high quality (Jadad score ≥ 3) and the remaining 4 as low quality (Jadad score < 3) (Table 1). The quality of evidence in GRADE analyses for each outcome ranged from ‘‘low” (22.2%) to “moderate” (44.4%) to ‘‘high” (33.3%) (S1 Table).

### Improvement of psychiatric symptoms

#### Primary outcome

**Fig 2:**
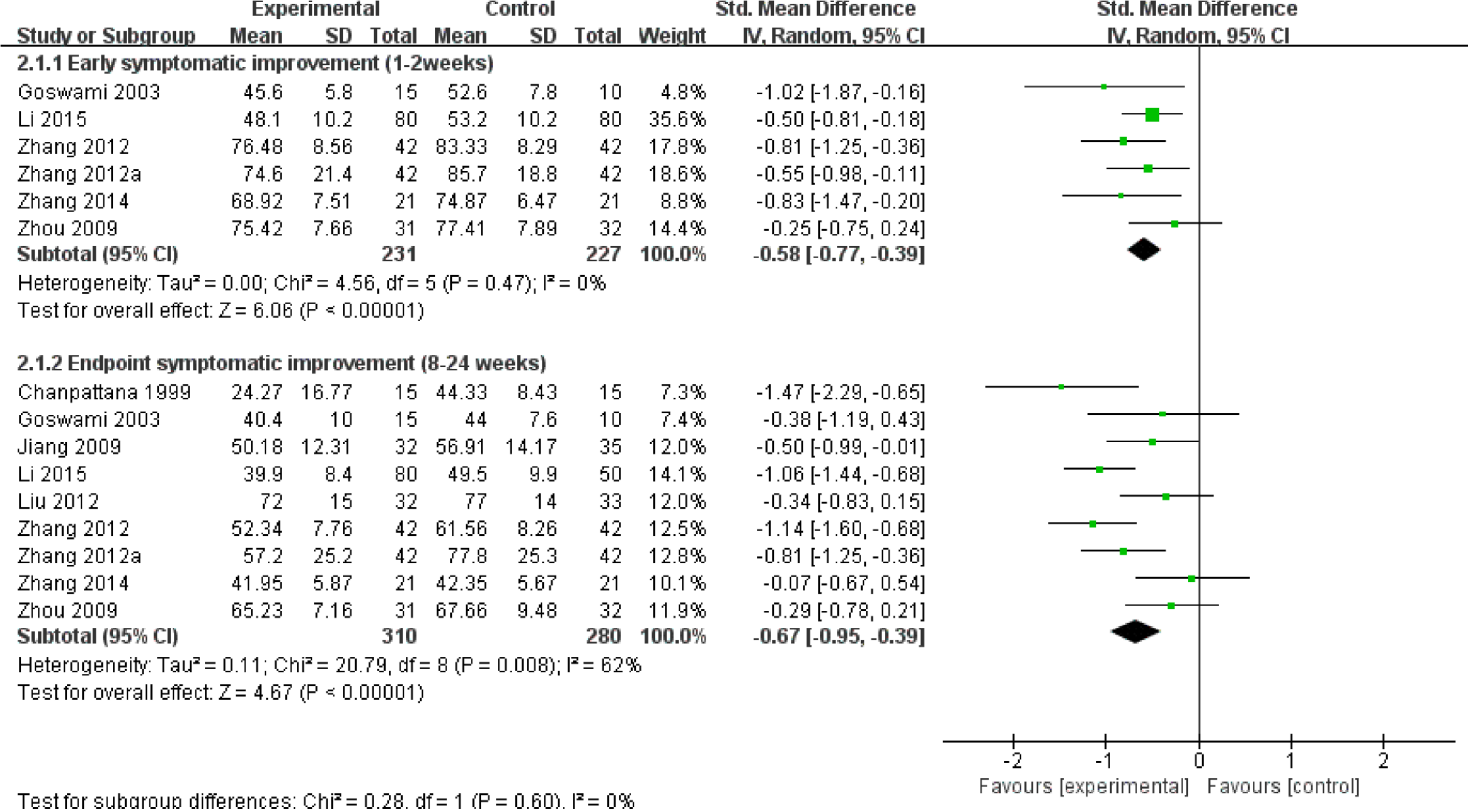
ECT added to non-clozapine antipsychotic medications for treatment-resistant schizophrenia: improvement in total psychopathology at 1–2 weeks and study endpoint.

**Fig 3:**
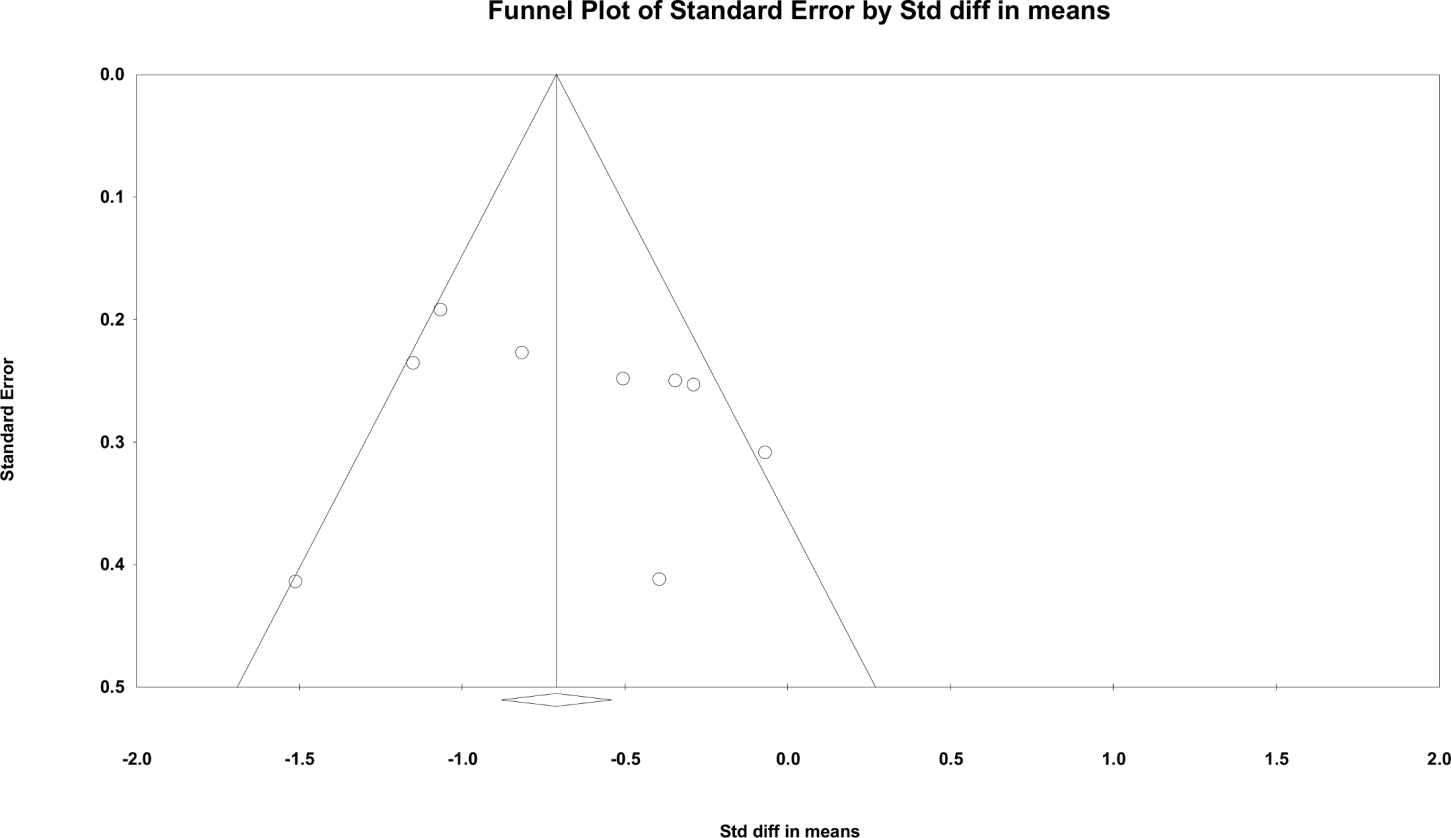
ECT added to non-clozapine antipsychotic medications for treatment resistant schizophrenia: publication bias.

Fig 2 demonstrates that the adjunctive ECT-antipsychotic combination outperformed the comparator on endpoint symptomatic improvement of the total scores of PANSS (6 trials) or BPRS (3 trials) with a SMD of -0.67 [(CI:-0.95,-0.39) (p<0.00001; I^2^ = 62%)]. The results remained consistent when the three outlier RCTs [14, 19, 23] were removed resulting in a SMD of -0.44 [(95%CI:-0.65, -0.22) (P<0.0001; I^2^ = 0%)]. Furthermore, added-on ECT was significantly superior in all but one preplanned subgroup analyses regarding endpoint symptomatic status (Table 2). In the exploratory meta-regression analyses, there were non-significant moderating effects on the endpoint symptomatic improvement including sample size (p = 0.25), mean age (p = 0.52), illness duration (p = 0.63), Jadad score (p = 0.28), trial duration (p = 0.56) and proportion of male patients (p = 0.46). The funnel plot was symmetrical (Fig 3), and the Egger’s test did not identify publication bias (p = 0.58). The fail-safe method indicated that an additional 134 studies would result in a negative result.

**Table 2:** Subgroup and sensitivity analysis of the effect of mediator variables on the outcome of “endpoint symptomatic improvement”.

Variables	Subjects (studies)	SMDs (95%CI)	I^2^ (%)	P
1. Chinese studies	535 (7)	-0.63 (-0.93, -0.33)	64	**<0.0001**
Non-Chinese studies	55 (2)	-0.92 (-1.99, 0.14)	0	0.09
2. Double blind/rater-masked	271 (5)	-0.74 (-1.16, -0.33)	61	**0.0005**
Non-blinded	319 (4)	-0.59 (-1.04, -0.15)	72	**0.009**
3. Trial duration < 12 weeks	323 (4)	-0.95 (-1.20, -0.70)	8	**<0.00001**
Trial duration ≥ 12weeks	267 (5)	-0.46 (-0.82, -0.10)	51	**0.01**
4. the number of ECT^a^: mean <9 sessions	84 (1)	-0.81 (-1.25, -0.36)	NA	**0.0004**
mean ≥9 sessions	481 (7)	-0.68 (-1.03, -0.33)	70	**0.0002**
5. High quality (Jadad score ≥ 3)	313 (6)	-0.63 (-1.03, -0.23)	64	**0.002**
Low quality (Jadad score < 3)	277 (3)	-0.74 (-1.18, -0.31)	66	**0.0009**
6. Co-starting with an antipsychotic	237 (4)	-0.49 (-0.75, -0.23)	1	**0.0003**
Augmenting with an antipsychotic	353 (5)	-0.83 (-1.26, -0.41)	70	**0.0001**

^a^Only 8 RCTs reported the number of ECT sessions. Bold values are p<0.05

CI: 95% confidence interval; ECT = electroconvulsive therapy; SMDs = standardized mean differences; NA = not applicable

#### Secondary outcomes

**Fig 4:**
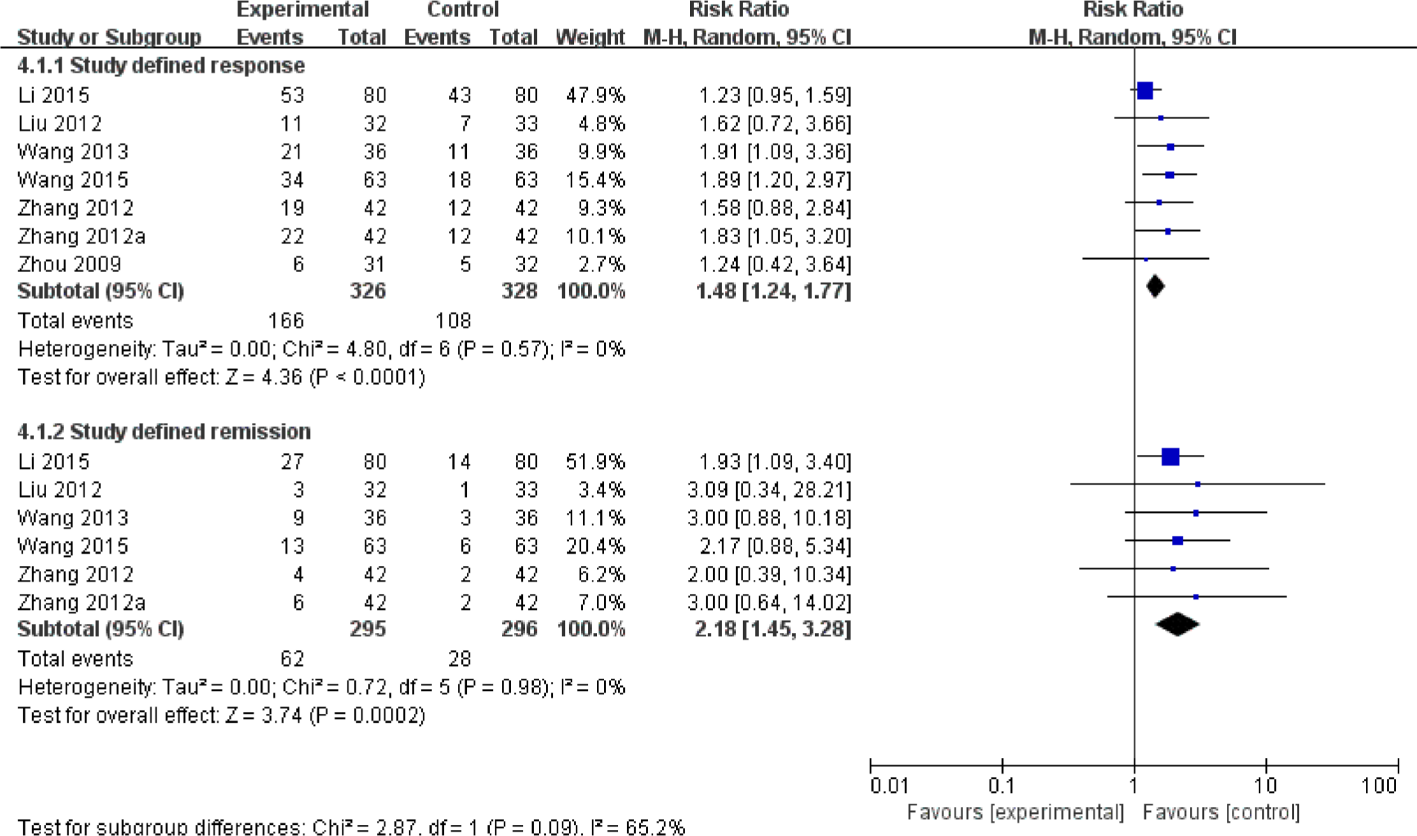
ECT added to non-clozapine antipsychotic medications for treatment resistant schizophrenia: study-defined response and remission.

Fig 2 and Fig 4 illustrate that adjunctive ECT outperformed the comparator on early symptomatic improvement in the total scores of PANSS (6 trials) or BPRS (2 trials) at 1 to 2 weeks with a SMD of -0.58 [(CI:-0.77, -0.39) (p<0.00001; I^2^ = 0%)] and for study-defined response (reduction in PANSS or BPRS total scores ≥50%) (RR = 1.48, p<0.0001) with a NNT of 6 (CI = 4–9) and remission (reduction in PANSS or BPRS total scores h a SM(RR = 2.18, p = 0.0002) with a NNT of 8 (CI = 6–16). Similar results were observed in terms of endpoint of PANSS positive and general symptom sub-scores with a WMD between -3.48 to -1.32 (P = 0.01 to 0.009), but not with respect to the PANSS negative symptom sub-score with a WMD of -1.01 (P = 0.17) (S4 Fig).

#### ADRs and discontinuation

Headache (RR = 5.35, p = 0.02) with a NNH of 6 (CI = 4–11) and memory impairment (RR = 14.30, p = 0.001) with a NNH of 3 (CI = 2–5) were significantly more frequent with ECT-antipsychotic co-treatment over antipsychotic monotherapy (S5 Fig). In one study [16] the number of discontinuation due to ADRs was 2 and 0 in the ECT-antipsychotic co-treatment and antipsychotic monotherapy groups, respectively. The remaining RCTs did not report all-cause discontinuation rate.

## Discussion

In this meta-analysis of 11 RCTs (n = 818) comparing add-on ECT to non-clozapine antipsychotics with antipsychotic monotherapy for TRS, the combination treatment was superior in terms of the primary and key secondary efficacy outcomes. Importantly, adjunctive ECT was both safe and well tolerated.

In terms of the primary outcome measurement, adjunctive ECT was significantly superior to antipsychotic monotherapy with a medium effect size; according to Cohen [41] criteria, SMD of –0.2, –0.5, and –0.8 were defined as small, medium and large effect size, respectively. While the heterogeneity of the primary outcome result was 62%, the resultant effect size was still -0.44 and heterogeneity decreased to 0% after conducting a sensitivity analyses. The result was the same across all but one *a priori* defined subgroup analyses, namely non-Chinese studies. Subgroup analyses did not reveal a significant difference between the Chinese (SMD = -0.63, 95%CI:-0.93, -0.33) and non-Chinese studies (SMD = -0.92, 95%CI:-1.99, 0.14). In meta-regression analyses, pre-specified baseline moderators or mediators of primary outcomes could not be identified. Publication bias was not detected by the funnel plot and Egger’s test.

Adjunctive ECT was significantly superior to antipsychotic monotherapy with a moderate effect size of -0.58 after 1 to 2 weeks, which increased to the larger effect size of -0.67 after 8 weeks suggesting an overall reliability of the results. The majority of patients responded better to adjunctive ECT than to antipsychotic monotherapy: 50.9% vs. 32.9%, NNT = 6. The remission rates similarly favored adjunctive ECT: 21.0% vs. 9.5%, NNT = 8. While the optimal number of ECT sessions for schizophrenia remains unclear, 12 to 20 sessions have been shown to be adequate [42, 43]. The number of ECT sessions were 14.2±8.0 (range = 7.6–36.0, median = 8.0) in ten studies. Thus, an ECT course of 14 sessions appears to be reasonable to target the symptoms of schizophrenia. Of note, in seven of the eleven studies, the electrode placement was reported. In all seven of these studies, bilateral placement was utilized.

Adjunctive ECT was generally safe and well-tolerated. Sixteen patients reported headache (18.3% vs. 2.2% on antipsychotic monotherapy, NNH = 6) and 32 experienced memory impairment in the adjunctive ECT group (34.3% vs. 1.5% on antipsychotic monotherapy, NNH = 3), which were consistent with Wang et al's study [25]. These ADRs were mostly mild, transient and tolerable [17, 18].

In a recent meta-analysis on adjunctive ECT combined with any type of antipsychotics for TRS (22 RCTs), 11 RCTs focused on concurrent use of ECT with non-clozapine antipsychotics [25]. However, the authors did not examine the separate effect of this strategy. Another systematic review concluded that ECT may be an effective and safe augmentation strategy to clozapine in TRS, but the effects of ECT combined with non-clozapine were not examined [12].

Several limitations of this meta-analysis need to be mentioned. First, blinding methods for raters were only reported in 4 studies and only 7 of the 11 RCTs were rated as high quality. Second, there was statistical heterogeneity of endpoint symptomatic improvement resulting from methodological and clinical heterogeneity. This limitation was partly offset by one sensitivity analysis, 5 subgroup analyses and 7 meta-regression analyses to identify potential moderators or mediators of the effect on primary outcome. Third, most studies had relatively short duration (median = 8.0 weeks). Fourth, although serious adverse effects, such as confusion, could occur after ECT, apart from the ADRs mentioned above, no serious adverse effects were reported in the studies. Finally, few objective measures evaluated neurocognitive functioning. Only 2 RCTs [17, 24] tested cognitive functions with the Wechsler Memory Scale without data for the control group in one RCT [24]; 1 RCT [14] applied the modified Mini-Mental State Examination; one RCT [16] used the Wisconsin Card Sorting Test.

## Conclusions

This systematic review and meta-analysis of 11 RCTs with 818 patients concluded that ECT added to a non-clozapine antipsychotic medication for TRS is more effective than antipsychotic monotherapy. The ECT/non-clozapine antipsychotic combination is safe and tolerable. Future studies should examine clinical factors that predict efficacy and tolerability of ECT for patients with schizophrenia.

## Supporting Information

S1 Fig PRISMA 2009 Checklist. (DOCX)

S2 Fig The excluded publications. (DOCX)

S3 Fig Risk of bias. (DOCX)

S4 Fig ECT added to non-clozapine antipsychotic medications for treatment resistant schizophrenia: the Positive and Negative Syndrome Scale (PANSS) positive, negative and general psychopathology sub-scores. (DOCX)

S5 Fig ECT added to non-clozapine antipsychotic medications for treatment resistant schizophrenia: adverse drug reactions. (DOCX)

S1 Table GRADE analyses: ECT added to non-clozapine antipsychotics for treatment resistant schizophrenia. (DOCX)
