# Supplementary material for: Electroconvulsive Therapy Added to Non-Clozapine Antipsychotic Medication for Treatment Resistant Schizophrenia: Meta-Analysis of Randomized Controlled Trials
Source: PLoS One. 2016 Jun 10;11(6):e0156510. doi: 10.1371/journal.pone.0156510 (PMC4902215; doi:10.1371/journal.pone.0156510)
Supplement: S2 Fig — (DOCX) [file pone.0156510.s002.docx]

S5 Fig. The excluded publications

1. Yang KJ, Liu TB, Yang HC, Gao H, Wu DH, et al. (2005) The effect of clozapine combining MECT for the resistant schizophrenics with agitation and aggressive behavior [In Chinese]. Medical Journal of Chinese People's Health 17: 485-486.

2. Wu DS, Gong M, Shang ZM, Chen FH (1992) A comparative clinical study on memory disfunctions induced by ECT [In Chinese]. Journal of Taishan Medical College 13: 280-282.

3. Wu BS, Liu QZ, Zhou HL, Liu SY, Xu CW, et al. (1989) A masked-rater randomized controlled trials of chlorpromazine combined with MECT versus chlorpromazine monotherapy for schizophrenia [In Chinese]. Chin J Nerv Ment Dis 15: 26-28.

4. WK T, GS U (2003) Efficacy of electroconvulsive therapy in treatment-resistant schizophrenia: a prospective open trial [In Chinese]. Progress in Neuro-Psychopharmacology &amp;amp; Biological Psychiatry 27: 373-379.

5. Wang ZM, Zhu H, Pan YL, Chiu HF, Correll CU, et al. (2015) Electroconvulsive therapy and its association with demographic and clinical characteristics in Chinese psychiatric patients. J ECT 31: 114-118.

6. Wang YB, Liu HW, Xia J (2012) The effect of ECT for patients with schizophrenia with Stupor behavior [In Chinese]. Contemporary Medicine 18: 79-80.

7. Wang WZ, Pu CC, Jiang JL, Cao QY, Wang JJ, et al. (2015) Efficacy and safety of treating patients with refractory schizophrenia with antipsychotic medication and adjunctive electroconvulsive therapy: a systematic review and meta-analysis [In Chinese]. Shanghai Archives of Psychiatry 27: 206-219.

8. Wang SS, Cao CB, Gu DH, Wang D (2014) Control study of MECT combined with small-dose aripiprazole in treatment of 120 cases with schizophrenia [In Chinese]. Medical Journal of Chinese People's Health 26: 3-6.

9. Wang GK (2015) Efficacy of ziprasidone combined with MECT in the treatment of refractory schizophrenia [In Chinese]. Medical Information 28: 43-43.

10. Ukpong DI, Makanjuola RO, Morakinyo O (2002) A controlled trial of modified electroconvulsive therapy in schizophrenia in a Nigerian teaching hospital. West Afr J Med 21: 237-240.

11. Tharyan P, Adams CE (2005) Electroconvulsive therapy for schizophrenia. Cochrane Database Syst Rev: CD000076.

12. Taylor P, Fleminger JJ (1980) ECT for schizophrenia. Lancet 1: 1380-1382.

13. Tang YL, Jiang W, Ren YP, Ma X, Cotes RO, et al. (2012) Electroconvulsive therapy in China: clinical practice and research on efficacy. J ECT 28: 206-212.

14. Tang WK, Ungvari GS, Leung HC (2002) Effect of piracetam on ECT-induced cognitive disturbances: a randomized, placebo-controlled, double-blind study [In Chinese]. J ECT 18: 130-137.

15. Tang WK, Ungvari GS (2003) Efficacy of electroconvulsive therapy in treatment-resistant schizophrenia: a prospective open trial. Prog Neuropsychopharmacol Biol Psychiatry 27: 373-379.

16. Tang WK, Ungvari GS (2002) Efficacy of electroconvulsive therapy combined with antipsychotic medication in treatment-resistant schizophrenia: a prospective, open trial. J ECT 18: 90-94.

17. Tang CD, Shen XL (2011) Cost-effectiveness analysis of MECT and risperridone on the treatment of schizophrenia [In Chinese]. Strait Pharmaceutical Journal 23: 181-183.

18. Sun HX, Mao YF, Shen YM, Dai XH (2015) Effects of no convulsions electric shock treatment on cognitive function in patients with schizophrenia [In Chinese]. China Modern Doctor 53: 68-72.

19. Song Y (2011) The effect of ECT for schizophrenia patients with the action of refusing food and drugs [In Chinese]. Medical Journal of Chinese People's Health 23: 584-585.

20. Small J, Milstein V, Klapper M, Kellams J, Small I (1982) ECT combined with neuroleptics in the treatment of schizophrenia. Psychopharmacol Bull 18: 34-35.

21. Shen YF (2009) Electroconvulsive therapy in Psychiatry (In Chinese). Beijing: People's Medical Publishing House.

22. Shen T, Wang B, Li C, Wang HF, Wang XL, et al. (2011) The effective study of olanzapine and MECT in acute schizophrenia patients [In Chinese]. Sichuan Mental Health 24: 89-91.

23. Sensky T, Turkington D, Kingdon D, Scott JL, Scott J, et al. (2000) A randomized controlled trial of cognitive-behavioral therapy for persistent symptoms in schizophrenia resistant to medication. Arch Gen Psychiatry 57: 165-172.

24. Sarkar P, Andrade C, Kapur B, Das P, Sivaramakrishna Y, et al. (1994) An exploratory evaluation of ECT in haloperidol-treated DSM-IIIR schizophreniform disorder. Convuls Ther 10: 271-278.

25. Sarita EP, Janakiramaiah N, Gangadhar BN, Unterrainer AF (1998) Efficacy of Combined ECT after Two Weeks of Neuroleptics in Schizophrenia: A Double Blind Controlled Study. Nimhans Journal 16: 243–251.

26. Sajatovic M, Meltzer HY (1993) The Effect of Short-Term Electroconvulsive Treatment Plus Neuroleptics in Treatment-Resistant Schizophrenia and Schizoaffective Disorder. Journal of Ect 9: 167-175.

27. Rao SX, Tan W (2014) Comparative Study on the Clinical Effects of Risperidone and Modified Electroconvulsive Therapy for Refractory Schizophrenia [In Chinese]. Chinese Journal of Pharmacovigilance 11: 580-583.

28. Petrides G, Malur C, Braga RJ, Bailine SH, Schooler NR, et al. (2015) Electroconvulsive therapy augmentation in clozapine-resistant schizophrenia: a prospective, randomized study. Am J Psychiatry 172: 52-58.

29. Peng CG, Wang GH, Zhang XF, Tu ZM, Cai DM, et al. (2014) Effect of MECT combined with risperidone oral Solution on irritability and agitation syndrome during the acute phase of schizophrenia [In Chinese]. Journal of Psychiatry 27: 427-429.

30. Pavlicevic M (1994) Improvisational music therapy and the rehabilitation of persons suffering from chronic schizophrenia. Journal of Music Therapy 31: 86-104.

31. Painuly N, Chakrabarti S (2006) Combined use of electroconvulsive therapy and antipsychotics in schizophrenia: the Indian evidence. A review and a meta-analysis. J ECT 22: 59-66.

32. Milstein V, Small JG, Miller MJ (1990) Mechanisms of action of ECT: Schizophrenia and schizoaffective disorder. Biological Psychiatry 27: 1282–1292.

33. Liu TZ, Wang SX, Wang B, Zhao GF, Wang LP, et al. (2008) A comparative study of modified electroconvulsive therapy for catatonic schizophrenia [In Chinese]. Journal of Psychiatry 21: 32-34.

34. Liu LZ (2010) Modified electric convulsive therapy on consolidation of schizophrenia patients [In Chinese]. Medical Journal of Liaoning 24: 71-72.

35. Li Y, Wu RQ, Sun FL (2014) Effects of modified electroconvulsive therapy on outcome and cognitive function of patients with schizophrenia [In Chinese]. Sichuan Mental Health 27: 418-421.

36. Li H, Chen XF (2009) A controlled study of modified electric convulsive therapy in negative symptoms of schizophrenia [In Chinese]. Linchuang Xinshengjibing Zazhi 15: 220-221.

37. König P, Glatter-Götz U (1990) Combined electroconvulsive and neuroleptic therapy in schizophrenia refractory to neuroleptics. Schizophrenia Research 3: 351-354.

38. Klein E, Kolsky Y, Puyerovsky M, Koren D, Chistyakov A, et al. (1999) Right prefrontal slow repetitive transcranial magnetic stimulation in schizophrenia: a double-blind sham-controlled pilot study. Biological Psychiatry 46: 1451–1454.

39. Jiang XQ, Yang KR, Zhou B, Jing P, Zheng LF, et al. (2009) Study on efficacy of modified electroconvulsive therapy(MECT)together with risperidone in treatment treatment-resistant schizophrenia(TRS) [In Chinese]. Chinese Journal of Nervous and Mental Diseases 35: 79-83.

40. Ji JJ (2011) A comparsion study of MECT and risperridone in the treatment of patients with treatment refractory schizophrenia [In Chinese]. Jilin Medical Journal 32: 7725.

41. Janakiramaiah N, Subbakrishan DK (1981) ECT-chlorpromazine combination compared with chlorpromazine only in schizophrenia. Indian J Psychiatry 23: 230-233.

42. Janakiramaiah N, Channabasavanna SM, Murthy NS (1982) ECT/chlorpromazine combination versus chlorpromazine alone in acutely schizophrenic patients. Acta Psychiatr Scand 66: 464-470.

43. Huang WS, Di XL, Ynag PD, Niu YJ, Ji ZF, et al. (2004) A comparative study of the effect of modern electroconvulsive therapy on the agitated behavior of psychotic patients [In Chinese]. Journal of Clinical Psychological Medicine 14: 276-277.

44. Hirose S, Ashby CR, Mills MJ (2001) Effectiveness of ECT combined with risperidone against aggression in schizophrenia. Journal of Ect 17: 22-26.

45. He LT (2014) The clinical effect of quetiapine combined with non convulsively electroconvulsive therapy in the treatment of female refractory schizophrenia [In Chinese]. Chinese Community Doctors 30: 79-80.

46. He JP (2011) A comparative study of antipsychotic combined with MECT for schizophrenia with agitation behaviors [In Chinese]. Medical Journal of Chinese People's Health 23: 2930-2931.

47. Han CL, Liu B (2011) Influence of multiparameter monitoring of MECT influence on event-related potential (ERP) of schizophrenia [In Chinese]. Zhejiang Clinical Medical Journal 13: 760-763.

48. Guo HM (2009) A control study of MECT in the treatment of agitated behavior of schizophrenia [In Chinese]. Journal of Clinical Psychosomatic Diseases 15: 488-489.

49. Gujavarty K, Greenberg LB, Fink M (1987) Electroconvulsive Therapy and Neuroleptic Medication in Therapy-Resistant Positive-Symptom Psychosis. Journal of Ect 3: 185-195.

50. Gao J, Zhang W (2009) Modified electroconvulsive treatment of refractory schizophrenia [In Chinese]. Modern Pharmacy and Clinic 24: 372-374.

51. Friedel RO (1986) The combined use of neuroleptics and ECT in drug resistant schizophrenic patients. Psychopharmacol Bull 22: 928-930.

52. Fink M (2001) Convulsive therapy: a review of the first 55 years. J Affect Disord 63: 1-15.

53. Du GP, Zhou GQ, Guan JH, Sun QY (2009) A comparative study of Quetiapine added with MECT in the treatment of excitement of schizophrenia [In Chinese]. Medical Journal of Chinese People's Health 21: 2985-2986.

54. Dodwell D, Goldberg D (1989) A Study of Factors Associated with Response to Electroconvulsive Therapy in Patients with Schizophrenic Symptoms. British Journal of Psychiatry 154: 635-639.

55. Chen YJ, Song BF, Wu Y (2013) A comparative study of the effect of modified electroconvulsive therapy and haloperidolin injection on patients with acute phase schizophrenic [In Chinese]. Sichuan Mental Health 26: 100-102.

56. Chen YF (2012) The effect of risperidone oral solution combining MECT for patients with schizophrenics with agitation and aggressive behavior [In Chinese]. Guide of China Medicine 10: 494-495.

57. Chen J, Liu SB. A control study of MECT and clozapien in the treatment of schizophrenia patients [In Chinese]; 1999; China. pp. 1.

58. Chen FY, Hou ZH, Wang ZH (2012) Effects of ziprasidone combined with modified electroconvulsive therapy on cognitive function of schizophrenia patient [In Chinese]. Linchuang Xinshengjibing Zazhi 18: 533-536.

59. Chanpattana W, Kramer BA (2003) Acute and maintenance ECT with flupenthixol in refractory schizophrenia: sustained improvements in psychopathology, quality of life, and social outcomes. Schizophrenia Research 63: 189–193.

60. Chanpattana W, Chakrabhand MLS (2001) Factors Influencing Treatment Frequency of Continuation ECT in Schizophrenia. Journal of Ect 17: 190-194.

61. Chanpattana W, Chakrabhand MLS (2001) Combined ECT and neuroleptic therapy in treatment-refractory schizophrenia: prediction of outcome. Psychiatry Research 105: 107-115.

62. Chanpattana W, Chakrabhand ML, Kongsakon R, Techakasem P, Buppanharun W (1999) Short-term effect of combined ECT and neuroleptic therapy in treatment-resistant schizophrenia. J ECT 15: 129-139.

63. Chanpattana W, Buppanharun W, Raksakietisak S, Chakrabhand MLS (2000) Seizure threshold rise during electroconvulsive therapy in schizophrenic patients. Psychiatry Research 96: 31-40.

64. Chanpattana W (2000) Maintenance ECT in treatment-resistant schizophrenia. Journal of the Medical Association of Thailand = Chotmaihet thangphaet 83: 657-662.

65. Cai XY, Liang JL, Su Y, Zheng YM, Luo XF, et al. (2008) A comparison study of MECT combining clozapine in the treatment of treatment-resistant schizophrenia [In Chinese]. Zhongguo Minkang Yixue 20: 1423-1424.

66. Bulbul F, Copoglu US, Alpak G, Unal A, Demir B, et al. (2013) Electroconvulsive therapy in pregnant patients. Gen Hosp Psychiatry 35: 636-639.

67. Brandon S, Cowley P, McDonald C, Neville P, Palmer R, et al. (1985) Leicester ECT trial: results in schizophrenia. Br J Psychiatry 146: 177-183.

68. Braga RJ, Petrides G (2005) The combined use of electroconvulsive therapy and antipsychotics in patients with schizophrenia. J ECT 21: 75-83.

69. Agarwal A, Winny GC (1985) Role of ect phenothiazine combination in schizophrenia. Indian J Psychiatry 27: 233-236.

70. Abraham KR, Kulhara P (1987) The efficacy of electroconvulsive therapy in the treatment of schizophrenia. A comparative study. Br J Psychiatry 151: 152-155.
