# Supplementary figures and images for: Electroconvulsive Therapy Added to Non-Clozapine Antipsychotic Medication for Treatment Resistant Schizophrenia: Meta-Analysis of Randomized Controlled Trials
Source: PLoS One. 2016 Jun 10;11(6):e0156510. doi: 10.1371/journal.pone.0156510 (PMC4902215; doi:10.1371/journal.pone.0156510)

**S1 Fig. Risk of bias**


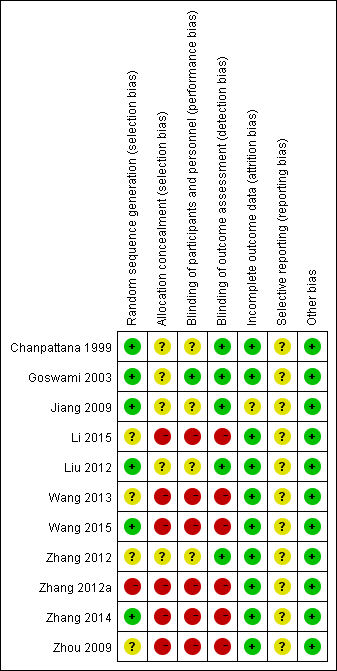

Supplement: S3 Fig — (DOCX) [file pone.0156510.s003.docx]
