# Supplementary material for: Electroconvulsive Therapy Added to Non-Clozapine Antipsychotic Medication for Treatment Resistant Schizophrenia: Meta-Analysis of Randomized Controlled Trials
Source: PLoS One. 2016 Jun 10;11(6):e0156510. doi: 10.1371/journal.pone.0156510 (PMC4902215; doi:10.1371/journal.pone.0156510)
Supplement: S4 Fig — (DOCX) [file pone.0156510.s004.docx]

**S2 Fig. ECT added to non-clozapine antipsychotic medications for treatment resistant schizophrenia: the Positive and Negative Syndrome Scale (PANSS) positive, negative and general psychopathology sub-scores**

**
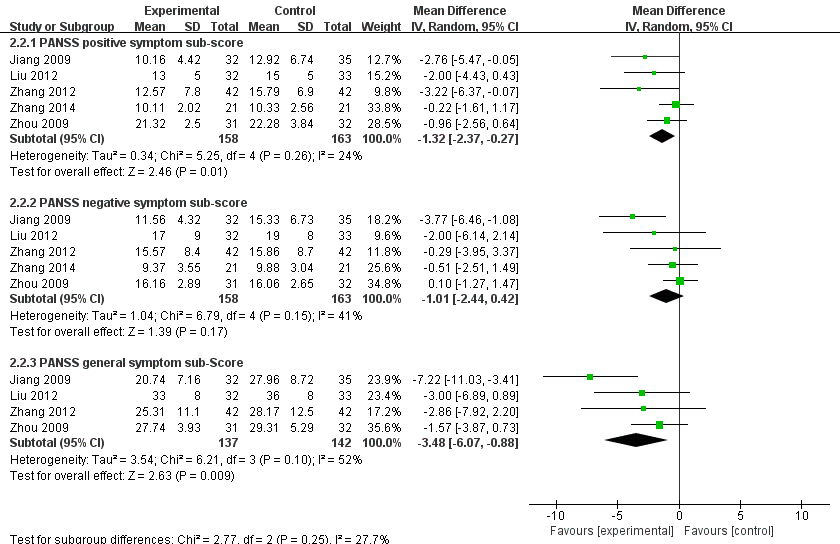
**
