# Supplementary material for: Electroconvulsive Therapy Added to Non-Clozapine Antipsychotic Medication for Treatment Resistant Schizophrenia: Meta-Analysis of Randomized Controlled Trials
Source: PLoS One. 2016 Jun 10;11(6):e0156510. doi: 10.1371/journal.pone.0156510 (PMC4902215; doi:10.1371/journal.pone.0156510)
Supplement: S1 Table — (DOCX) [file pone.0156510.s006.docx]

**S1 Table. GRADE analyses: ECT added to non-clozapine antipsychotics for treatment resistant schizophrenia**

| **Primary/*secondary* outcomes^a^** | **N**^b^  **(studies)** | **Risk of bias**^c^ | **Inconsistency**^d^ | **Indirectness** | **Imprecision** | **Publication bias** | **Large effect**^e^ | **Is it plausible that confounding factors would change the effect?** | **Dose response gradient** | **Overall quality of evidence**^f^ |
| --- | --- | --- | --- | --- | --- | --- | --- | --- | --- | --- |
| **Total Psychopathology at Study Endpoint** | 590 (9) | Serious | Serious | No | No | Undetected | No | No | No | +/+/-/-/; Low |
| **Total Psychopathology at 1-2 Weeks** | 458 (6) | Serious | No | No | No | Undetected | No | No | No | +/+/+/-/; Moderate |
| ***Study-defined Response*** | 654 (7) | Serious | No | No | No | Undetected | No | No | No | +/+/+/-/; Moderate |
| ***Study-defined Remission*** | 591 (6) | Serious | No | No | No | Undetected | Large | No | No | +/+/+/+/; High |
| ***PANSS Positive Symptom Sub-Score*** | 321 (5) | Serious | No | No | No | Undetected | No | No | No | +/+/+/-/; Moderate |
| ***PANSS Negative Symptom Sub-Score*** | 321 (5) | Serious | No | No | No | Undetected | No | No | No | +/+/+/-/; Moderate |
| ***PANSS General Symptom Sub-Score*** | 279 (4) | Serious | Serious | No | No | Undetected | No | No | No | +/+/-/-/; Low |
| ***Memory Impairment*** | 135 (4) | Serious | No | No | No | Undetected | Very Large | No | No | +/+/+/+/; High |
| ***Headache*** | 267 (4) | Serious | No | No | No | Undetected | Very Large | No | No | +/+/+/+/; High |

^a^This row describes all studies reporting that **primary (in bold)** or ***secondary*** ***(in bold and italic)*** measure. Subgroups of studies from the same measure may be described in lower rows. ***ECT = electroconvulsive therapy; PANSS = Positive and Negative Syndrome Scale;***

^b^N=total number of individuals after adding all studies providing that outcome. The number of studies is described in parentheses.

^c^All studies reported as having a serious bias used a single-blind method and only mentioned random allocation without describing the method.

^d^All studies reported as having a serious inconsistency had I^2^ > 50%.

^e^Studies with large effects (or even more with very large) provided increased quality of evidence. Large effects= 2<RR<0.5. Very large effects= 5<RR<0.2.

^f^GRADE Working Group grades of evidence: High quality=further research is very unlikely to change our confidence in the estimate of effect. Moderate quality=further research is likely to have an important impact on our confidence in the estimate of effect and may change the estimate. Low quality=further research is very likely to have an important impact on our confidence in the estimate of effect and is likely to change the estimate. Very low quality=we are very uncertain about the estimate.
